# Changes in vegetation-water response in the Sahel-Sudan during recent decades

**DOI:** 10.1016/j.ejrh.2024.101672

**Published:** 2024-04

**Authors:** Tingting Lu, Wenmin Zhang, Christin Abel, Stéphanie Horion, Martin Brandt, Ke Huang, Rasmus Fensholt

**Affiliations:** Department of Geosciences and Natural Resource Management, University of Copenhagen, Copenhagen, Denmark

**Keywords:** Cumulative effect duration, Climate variability, Drylands, Time series analysis, Vegetation-water sensitivity

## Abstract

**Study region:**

The Africa Sahel-Sudan region, defined by annual rainfall between 150 and 1200 mm.

**Study focus:**

Understanding the mechanism of vegetation response to water availability could help mitigate the potential adverse effects of climate change on global dryland ecosystems. In the Sahel-Sudan region, spatio-temporal changes and drivers of the vegetation-water response remain unclear. This study employs long-term satellite water and vegetation products as proxies of water availability and vegetation productivity to analyze changes in vegetation-water sensitivity and the cumulative effect duration (CED) representing a measure of the legacy effect of the impact of water constraints on vegetation. A random forest model was subsequently used to analyze potential climatic drivers of the observed vegetation response.

**New hydrological insights for the region:**

During 1982–2016 we found a significant decrease (*p* < *0.05*) in the sensitivity of vegetation productivity to water constraints in 26% of the Sahel-Sudan region, while 9% of the area showed a significantly increased sensitivity, mainly in the sub-humid zone. We further showed that CED significantly increased and decreased, respectively in around 9% of the study area in both cases. Our climatic driver attribution analysis suggested the existence of varying underlying mechanisms governing vegetation productivity in response to water deficit across the Sahel-Sudan dryland ecosystems. Our findings emphasize the need for diverse strategies in sustainable ecosystem management to effectively address these varying mechanisms.

## Highlights

• A framework to study dryland vegetation-water response in relation to water deficit.
• Vegetation-water response is land-cover dependent in Sahel-Sudan during 1982-2016.
• Vegetation-water sensitivity is decreasing/increasing in 26%/9% of the region.
• CO_2_ and temperature primarily influence changes in the vegetation-water response.

## Graphical Abstract

**Figure:**
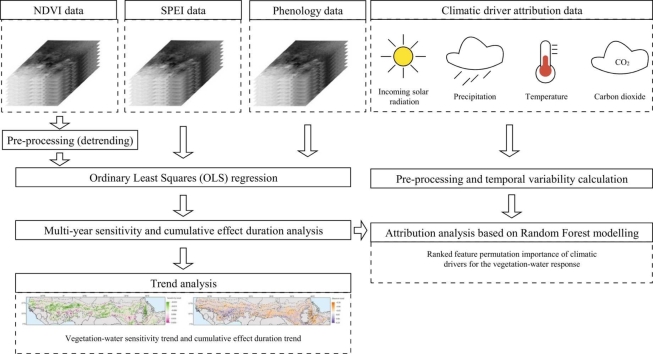

## 1 Introduction

A better understanding of vegetation-water interactions is of key interest in the field of ecohydrology, since vegetation represents a vital component of the hydrologic cycle. On the one hand, plants regulate water fluxes and energy exchange between the atmosphere and land surface through photosynthesis and evaporation (Tietjen et al., 2010, Zhang et al., 2022) and on the other hand, water availability controls plant growth and thus standing vegetation (Osakabe et al., 2014). The African Sahel-Sudan region is one of the largest dryland areas worldwide, and livelihoods are to a large extent characterized by rain-fed agriculture and pastoralism (Thébaud and Batterbury, 2001, Ramarohetra et al., 2013). The impacts of climate change on vegetation-water relations are likely not only to influence the ecosystem functioning of this region such as carbon sequestration potential and the water cycle (Brüggemann et al., 2011, Ogutu et al., 2021), but will also pose a threat to the food security of local communities (Connolly-Boutin and Smit, 2016). After severe droughts in the 1970 s and 1980 s, the Sahel-Sudan region experienced a re-greening, found to be primarily driven by an overall increase in precipitation (Brandt et al., 2015, Leroux et al., 2017, Pausata et al., 2020). This greening trend combined with recent warming may, however, result in an increasing water demand (Jiao et al., 2021, Zhang et al., 2022). In contrast, the general increase in precipitation observed over recent decades may weaken the susceptibility to the otherwise adverse impacts of water stress on vegetation growth (Fu, Shen and Zhang, 2018). These contrasted interactions between vegetation and water availability highlight the importance of gaining a better understanding of the mechanism of dryland vegetation-water response in ongoing global change.

Analysis of the vegetation-water response is well established through concepts like vegetation-water sensitivity and cumulative effect duration (CED) (Du et al., 2019, Abel et al., 2021, Jiao et al., 2021). Vegetation water sensitivity refers to the degree to which vegetation growth, photosynthesis, and productivity are impacted by changes in water availability. Sensitivity varies as a function of several aspects including differences in vegetation species and environmental factors such as the type of soil, climate, and topography (Norton et al., 2022). CED provides additional insight into the temporal aspects of vegetation water sensitivity. It measures the legacy effect of the impact of water constraints on vegetation, thereby representing the temporal extent during which vegetation has been affected by water constraints (Yao et al., 2022). Depending on site-specific conditions, CED can have long-lasting effects on the health and resilience of an ecosystem. The longer the CED, the more longer or severe the impact on vegetation can be, potentially leading to a decrease in productivity and changes in species composition, especially in arid regions (Guerschman et al., 2020, Shi et al., 2021). Despite several studies utilizing time series of vegetation and climatic data to examine the vegetation-water sensitivity and CED (Shi et al., 2021, Zhou et al., 2021, Wei et al., 2022), no attempts have been made to combine the sensitivity and CED into a unifying framework to study the temporal dynamics in the Sahel-Sudan region.

The vegetation-water relationship is closely related to the climate changes of a given location (Gan, Liu and Sun, 2021). Recent studies have also shown that climate variability such as changes in the seasonal distribution of rainfall could cause changes in dryland vegetation structure (Zhang et al., 2019, D'Adamo et al., 2021), and such changes may potentially influence the vegetation-water response. Yet, it remains unclear what is the impact of the temporal variability of climatic drivers on vegetation response to water availability and stress. In particular, there is currently some uncertainty concerning the direction of future climate change in the Sahel-Sudan region among various model predictions (Kent et al., 2015, Rowell et al., 2016), and an improved understanding of how vegetation is responding to water availability under climate change during the past decades would serve as a reference for investigations of future changes.

This study analyses the relationship between Normalized Difference Vegetation Index (NDVI) and Standardized Precipitation Evapotranspiration Index (SPEI) as proxies for vegetation growth and water availability, respectively, during the period covering 1982–2016. Furthermore, different climatic factors (temperature, precipitation, CO2, and solar radiation, as well as their temporal variability) are used to analyze how vegetation responds to changes in water availability. Specifically, the aims of this study are: (1) to understand the variations and changes in vegetation-water response in the Sahel-Sudan region over the past 35 years by analyzing vegetation-water sensitivity and CED and (2) to examine the relative importance of underlying climatic drivers to the observed trends in vegetation-water sensitivity and CED.

## 2 Material and methods

### 2.1 SPEI data

The SPEI is an index designed to quantify drought, and it enables the measurements of both wetness (positive value) and dryness (negative value). It is calculated as monthly observations for time integration periods of 1–48 months, where these different time scales are produced to characterize the cumulative water balance condition between potential evapotranspiration and precipitation including a given number of previous months from one to 48 months. SPEI data covering the Sahel-Sudan region over a 35-year period (1982–2016) with a spatial resolution of 0.05 degrees and a monthly time resolution (Peng et al., 2019) was used in this study. It is computed based on precipitation estimates from the satellite-based Climate Hazards group InfraRed Precipitation with Station data (CHIRPS) and potential evaporation estimates by the Global Land Evaporation Amsterdam Model (GLEAM). The ecosystems of the arid and semi-arid Sahel-Sudan region is characterized by a short rainy season of 3–5 months, followed by an extended dry season of 7–9 months, and the vegetation reacts primarily to water anomalies of the concurrent year (Vicente-Serrano et al., 2013, Ratzmann et al., 2016). Therefore, only SPEI data with a time integration scale of the previous ≤ 12 months were taken into consideration in this study, referred to as SPEI-1 to SPEI-12.

### 2.2 Vegetation productivity and phenology data

The NDVI data is derived from the NOAA Climate Data Record (CDR) of the AVHRR Normalized Difference Vegetation Index (Vermote, 2019), and it was used as a proxy for vegetation growth between 1982 and 2016. The NDVI time series consists of monthly composites according to the maximum value of the raw data to reduce different sources of errors related to atmospheric perturbations and sun-sensor viewing geometry. To eliminate the influence of seasonal variation, NDVI anomalies were calculated and applied in this study. Monthly NDVI anomalies have been computed by the following Eq. (1):

(1) $${\mathrm{NDVI}}_{\mathrm{anomaly}\;}={\mathrm{NDVI}}_{\mathrm{month},\mathrm{year}}-{\overset{®}{\mathrm{NDVI}}}_{\mathrm{mont}h}$$

Where ${\mathrm{NDVI}}_{\mathrm{anomaly}}$ is the NDVI anomaly, ${\overset{®}{\mathrm{NDVI}}}_{\mathrm{mont}h}$ is the long-term mean for this month from 1982 to 2016, ${\mathrm{NDVI}}_{\mathrm{month},\mathrm{year}}$ is the average NDVI measured for a month in a given year.

The re-greening of the region implies an increasing trend in the NDVI anomalies and to minimize its influence on analysis, linear detrending was subsequently performed on the monthly NDVI anomaly data. Linear detrending first finds the best-fitting straight line that represents the overall trend in the monthly NDVI anomalies data, by linear regression. Then, it subtracts the linear trend from the original data by subtracting the predicted values of the linear trend from the original data. The result is a detrended time series, where the linear trend has been removed.

The MCD12Q MODIS Land Cover Dynamics (LCD) product captures global land surface phenology at a 500 m resolution and has proven effective in examining various climate-ecosystem associations on a large spatial scale (Ganguly et al., 2010, Xie et al., 2022). The dataset comprises seven layers that document different phases of land surface vegetation phenology. As water availability during the early to the mid-growing season is key to vegetation growth (Zhang et al., 2019, Zhang et al., 2019), we defined the growing season to start the month before the occurrence of the green-up as observed in the LCD product and end with the month after the peak value. As the temporal coverage is limited to 2001 to present, we used per-pixel average values from 2001 to 2020 to represent the phenology of the study area. The monthly NDVI anomalies and SPEI data (SPEI-1 – SPEI-12) covering the growing season will be deployed in the following vegetation-water relationship analysis.

### 2.3 Climate and ancillary data

Monthly temperature data and incoming solar radiation data are obtained from ERA5-Land monthly averaged data (Muñoz-Sabater et al., 2021). Monthly precipitation data is aggregated from the daily CHIRPS data (Funk et al., 2015). Monthly carbon dioxide data is available from the US National Oceanic and Atmospheric Administration Earth System Research Laboratory (NOAA/ESRL) and collected by the Global Carbon Project. The 100 m land cover map is produced by the global component of the Copernicus Land Service (Buchhorn et al., 2020) in 2016. All datasets were subsequently resampled to the same resolution as the SPEI grid by averaging or using the majority value (land cover).

### 2.4 Vegetation water sensitivity and CED

To study the spatio-temporal distribution of vegetation-water sensitivity, an analysis was conducted on the relationship between water availability and vegetation growth during the growing season. Here, we use an Ordinary Least Squares regression (OLS) regression between observations of NDVI anomalies and SPEI during the growing season, and the r-value was subsequently used as a proxy for the vegetation-water sensitivity. A moving window of five consecutive years was used to provide a minimum amount of samples required for the OLS regression across time (Jenkins and Quintana-Ascencio, 2020).

OLS regression was performed to retrieve r-values between NDVI anomalies and SPEI for individual time scales from 1 to 12 months using a 5-year moving window approach. The final sensitivity of vegetation to water availability in a given year is determined by the r-value with the first peak or plateau between NDVI anomalies and SPEI, calculated over a period of 1–12 months (Supplementary Fig. 1).

For a grid cell to show a positive value in sensitivity, the NDVI anomaly is expected to increase/decrease with wetting/drying conditions, respectively. This collectively suggests that vegetation growth is constrained by water availability. On the contrary, a grid cell with a negative value in sensitivity follows from a situation where NDVI is decreasing with wetting conditions. Since water constraint is dominating in the drylands of the Sahel-Sudan region, we excluded regions with a negative sensitivity from the following analysis. Here, the water constrained region is defined as the area where the average r-value of the research period (1984–2014) is positive (note that the length of the time series is truncated by two years in the beginning and the end, due to the use of a 5-year moving window).

The temporal trend of the sensitivity was analyzed by the Mann-Kendall trend test for each grid cell in water constrained regions. A larger r-value denotes that vegetation is more vulnerable to water stress. An increasing trend thereby represents an increase in the sensitivity towards water constraints, whereas a decreasing trend denotes a decline in vegetation-water sensitivity. The land cover of the Sahel-Sudan region consists of shrubs (30%), herbaceous (29%), cropland (22%), forest (16%), and others (2%). The sensitivity trends among the four main landcover types is analyzed to further compare the variability in vegetation-water response.

The vegetation-water response is also analyzed by the use of the cumulative effect duration (CED) concept (Zhou et al., 2021). The SPEI at month n represents the current month and previous n-1 months water balance conditions. The CED is determined by calculating the correlation coefficient (r-value) between NDVI anomalies and SPEI across the months of the growing season using SPEI-1 to 12. Here the time-period with the highest r-value obtained, that is the first peak or plateau in r-values between NDVI anomalies and SPEI related to the use of SPEI from 1 to 12 months, is considered the CED (supplementary Fig. 1). The CED signifies the duration of which vegetation has been affected by water constraints. A larger value of CED typically suggests that the impact of water stress on vegetation is longer and/or more severe (Guerschman et al., 2020, Shi et al., 2021). We refrain from interpreting the CED in regards to severity and here only consider CED as a quantification of the duration of water stress. Larger values of CED are potentially to be expected in the southern part of the Sahel-Sudan region, as the growing season is longer as compared to the northern part. However, the per-pixel temporal trend in CED is not affected by this. Vegetation has various response mechanisms towards water constraints, and the CED value might be influenced by the vegetation physiology and eco-climatic conditions (Zhou et al., 2021). We therefore further analyzed the CED as a function of land cover classes. The temporal trend of CED was analyzed by the Mann-Kendall trend test at the grid cell level.

### 2.5 Attribution analysis of the vegetation-water sensitivity and CED

Various drivers could potentially influence the relationship between water availability and vegetation. We examined the impact of precipitation, temperature, incoming solar radiation, CO_2_, and their respective temporal variability on the vegetation-water sensitivity and CED by using a random forest (RF) model. Although precipitation and temperature are both related to data on water availability (SPEI), they were still considered for the analysis of the potential difference in the SPEI-related vegetation-water sensitivity and CED. The mean and standard deviation values of monthly precipitation, temperature, radiation, and CO_2_ in the growing season were applied to represent climatic conditions and their temporal variability. The climate data were averaged using a 5-year moving window to align them with the vegetation response data.

The RF model is an ensemble learning approach that integrates numerous decision tree models to generate predictions through the collective aggregation of their outputs (Breiman, 2001). Since the RF model in this study is used for an attribution analysis rather than a prediction, all the samples were used as the training set to ensure a model with the highest possible explanatory power. Specifically, 57090 observations were used for in the RF model training of the vegetation-water sensitivity attribution analysis and 29053 observations were used for the CED attribution analysis. The response variable of the RF model was the trend of vegetation-water sensitivity or CED over the period of investigation, and the independent variables were the trend of the climate variable features. The relative importance of each independent feature was compared by the permutation feature importance, which measures the reduction in model performance when a single feature value is randomly shuffled. In addition, a partial dependence analysis/plot for the ranked climatic drivers was used to show the effect of the individual features on the predicted outcome of the RF model. This analysis is restricted to the water constrained region, characterized by a significant (*p* < *0.05*) vegetation response trend.

## 3 Result

### 3.1 Spatio-temporal distribution of water sensitivity

**Fig. 1:**
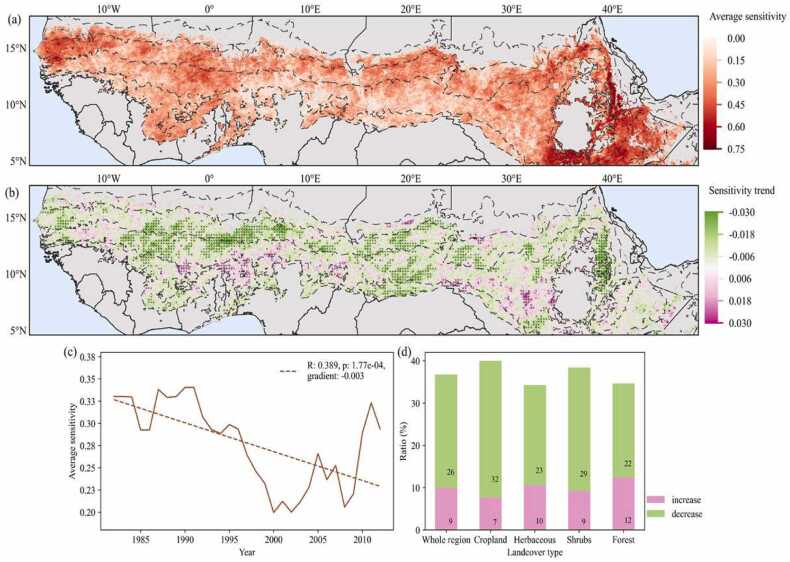
(a) Geographical patterns of average vegetation-water sensitivity during the period of analysis (1984–2014) and (b) the trend in sensitivity. The black dashed line represents the isohyet of 150, 300, 600, 1000 and 1200 mm; the black dots indicate a significant trend in sensitivity (*p < 0.05*). The regions not constrained by water (where the average r-value is negative) was masked out from (a) and (b). (c) Temporal dynamics of average sensitivity to water constraints and (d) the proportions of pixels of significant trends of vegetation-water sensitivity as a function of the four main land cover types.

According to the average vegetation-water sensitivity for the period 1984–2014, most of the Sahel-Sudan region (94%) is found to be water constrained (Fig. 1a). Spatially, higher precipitation is related to a lower average sensitivity value in the water constrained region, except for the sub-humid regions in East Africa. We analyzed the temporal trend of sensitivity at the pixel-level by the Mann-Kendall trend test, and results show that 35% of the research region is characterized by significant trends (*p < 0.05*) in vegetation-water sensitivity over the past three decades (Fig. 1b). Of these, we found that 9% of the region has become more sensitive to water constraints, and 26% has become less sensitive to water constraints (indicated by pink and green colors also including black dots in Fig. 1b). The appearance of decreasing sensitivity is mainly observed in the semi-arid zone where annual precipitation is between 300 and 600 mm, whereas, the area of increasing sensitivity is mainly found in the arid or wetter sub-humid area zone where precipitation is less than 300 mm and more than 800 mm (Supplementary Fig. 2a).

Generally, the annual average sensitivity shows a significant (*p < 0.05*) decreasing trend at the rate of 0.003 per year (Fig. 1c), however with a clear shift in trend observed around 2000. The period prior to 2000 is characterized by a clear decline in the annual average vegetation-water sensitivity over the Sahel-Sudan region; a tendency that is reverted in the subsequent period with the turn of the millennium marking a turning point in the results. Among the four main land cover types, croplands exhibit the highest proportion of areas showing a decrease in sensitivity, while they have the lowest proportion of areas of increasing sensitivity. Although forest on average shows the lowest sensitivity to water constraints (Supplementary Fig. 2b), it is also observed that forests show the largest proportion of areas displaying an increasing trend in sensitivity and the smallest proportion of areas showing a decreasing trend in sensitivity.

### 3.2 The spatio-temporal distribution of CED

**Fig. 2:**
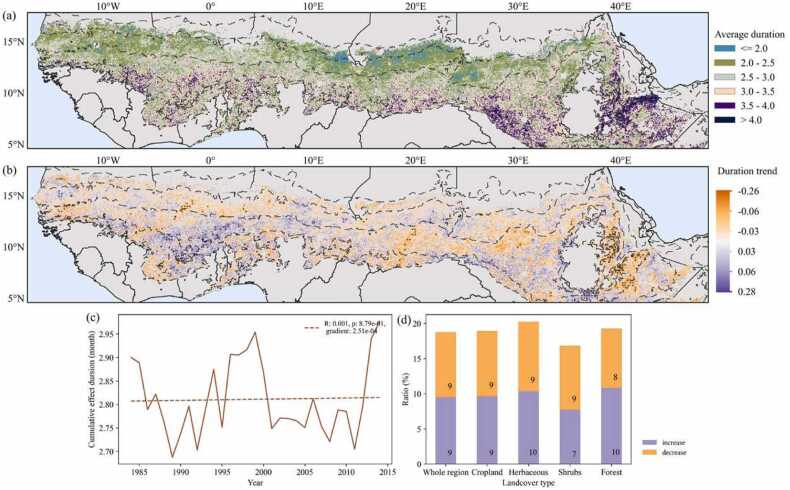
(a) Geographical patterns of average CED from 1984 to 2014 and (b) the trend in CED. The black dashed line represents the isohyets of 150, 300, 600, 1000 and 1200 mm; the black dots indicate a significant trend of CED with *p* < *0.05*. The regions not constrained by water (where the average r-value is negative) was masked out from (a) and (b). (c) Temporal dynamics of average CED values. (d) The proportion of pixels of increasing or decreasing trends in CED for different land cover classes.

Based on the time scales at which the sensitivity between NDVI anomalies and SPEI are recorded, this study found the CED in the water constrained Sahel-Sudan region to be mainly limited to 2–3 months, and the CED increases alongside the increased precipitation from north to south (Fig. 2a) causing also a longer growing season from north to south. In total 9% of the water constrained Sahel-Sudan region is characterized by a significant increase in CED, whereas 9% of the region shows a significant decrease in CED (indicated by purple and orange colors also including black dots in Fig. 2b). The ratio of pixels of significantly increasing CED along the precipitation gradient from dry to wet is gradually increasing and reaches peaks in the semi-arid and wetter sub-humid zones, whereas the ratio of pixels showing a decreasing trend in CED reaches a peak in the drier sub-humid zone (Supplementary Fig. 3a).

The average CED dynamics within the water-constrained region exhibit some temporal fluctuations, characterized by a minimal non-significant increase (*p > 0.05*, Fig. 2c). Furthermore, there are three distinct peaks in CED observed around the years 1984, 1998, and 2014, respectively. The proportion of areas showing a decreasing trend in CED among the four land cover types is all around 9%. On the contrary, the distribution of increasing trends varies, with forests and herbaceous areas having the highest proportions, while shrub areas exhibit the lowest proportion (Fig. 2d). The average CED during the past 3 decades is shortest for the herbaceous land cover class, followed by cropland, shrub, and forest (Supplementary Fig. 3b).

### 3.3 Attribution analysis

**Fig. 3:**
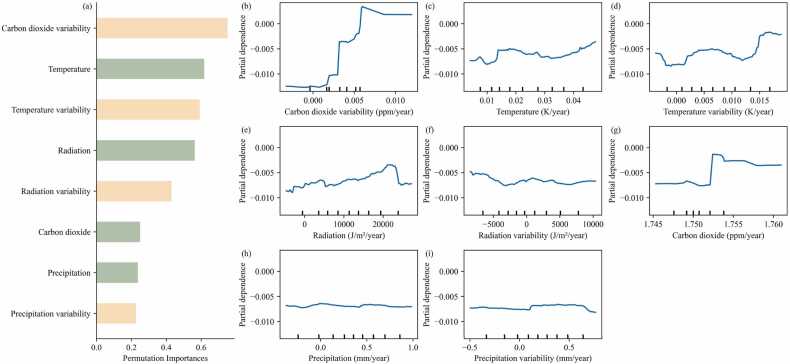
(a) The ranked feature permutation importance of climatic features for the trend in vegetation-water sensitivity. The green and yellow colors represent average climate features and climate variability features, respectively. (b)-(i) Partial dependence plots of the ranked climatic driver variables for the trend in vegetation-water sensitivity.

The relative contribution of climatic drivers on the vegetation-water sensitivity and CED dynamics was analyzed using RF models. The ranked permutation importance of each climatic variable in explaining the vegetation-water sensitivity trend in the water constrained Sahel-Sudan region showed that the CO_2_ variability has the largest impact on vegetation-water sensitivity, followed by temperature (mean and variability, Fig. 3a). The total amount of instances to train the RF model was 57090 and the R-square of the RF model was found to be 0.97.

The partial dependence of the CO_2_ variability on the predicted sensitivity showed that when CO_2_ variability increases, the predicted sensitivity trend turns positive and reaches a plateau (Fig. 3b). Due to the limited data points for CO_2_ variability exceeding values of 0.006 ppm/year, the partial dependence estimates in those regions may be less reliable. Additionally, increasing temperature (mean and variability) showed to have a positive effect on the sensitivity (Figs. 3c and 3d). A larger value of variability refers to more occurrences of climatic variations above or below normal conditions, which indicates that warmer or more extreme temperatures would potentially also increase vegetation-water sensitivity.

**Fig. 4:**
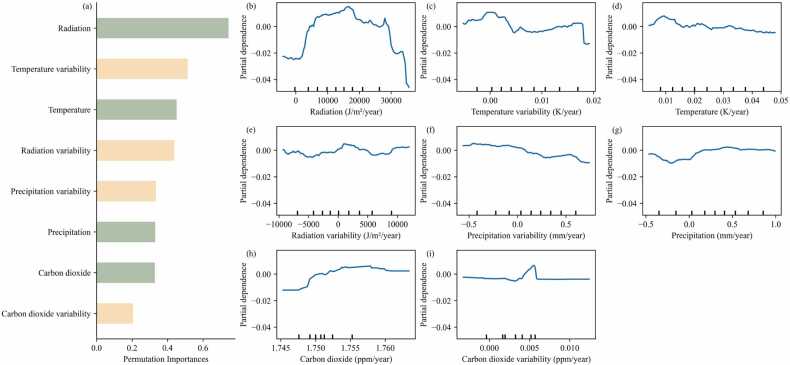
(a) The ranked feature permutation importance of climatic features for the trend in CED. The green and yellow colors represent the average climate feature and climate variability features, respectively. (b)-(i) Partial dependence plots of the ranked climatic driver variables for the trend in CED.

The total amount of instances to train the RF model on the CED dynamic was 29053, and the R-square of the RF model was found to be 0.94. Incoming solar radiation was found to be the most important feature for CED trends among climatic drivers, followed by temperature (mean and variability, Fig. 4a). An increase in incoming solar radiation leads to an increase in the CED trend until 20000 J/m^2^/year. Then, further increases in radiation correspond to a decrease in the CED trend (Fig. 4b). Similarly, higher average or variability in temperatures are associated with a reduction in CED duration (Fig. 4c and Fig. 4d). Furthermore, the partial dependence of other climatic drivers showed that their impact on the CED trend was limited (Fig. 4e-i).

## 4 Discussion

### 4.1 Spatio-temporal dynamics of vegetation-water sensitivity and CED

A higher sensitivity of vegetation to water variability was mostly observed in the regions with frequent drought occurrences as documented in previous research (Masih et al., 2014, Haile et al., 2019) following from the fact that drought represents a severe manifestation of water constraints that severely affects vegetation productivity. The observed overall decline in vegetation-water sensitivity supports the multiple findings of the vegetation recovery of the Sahel since the major droughts of the 80 s (Brandt et al., 2015, Leroux et al., 2017). After 2000, the declining trend reversed, but did not revert back to the levels of the 1980 s (Fig. 2d). This is in line with other scholars on the occurrence of droughts in the Sahel-Sudan countries after 2000 (Masih et al., 2014). Over the past 3 decades, 26% of all pixels in Sahel-Sudan characterized by being water constrained areas show a significant (*p < 0.05*) decrease in sensitivity, whereas only 9% show an increase in sensitivity mainly located in the arid and wetter sub-humid areas. The later coincides with areas in South Sudan, which have been subject to intensified and widespread prolonged droughts in recent two decades (Elagib and Elhag, 2011, Lyon, 2014). The land cover specific analysis showed that forests have a relatively smaller sensitivity value than herbaceous and shrubs. This might be related to the predominance of a woody stratum typically possessing deeper roots compared to the herbaceous stratum, enabling easier access to deeper soil water resources (Williams and Albertson, 2004, Yinglan et al., 2019). At the same time, the forest land cover class showed the largest proportion of areas displaying an increased sensitivity and the smallest proportion of areas showing a decreased sensitivity, which could be a warning sign of increasing vulnerability.

The result of the spatial distribution of average CED shows a relationship with the rainfall gradient, which is consistent with previous studies showing that rainfall is a determining factor for vegetation growth in these arid and semi-arid regions (Shi et al., 2021, Zhou et al., 2021, Zhang et al., 2022) and a spatial pattern of CED exists in the way vegetation growth responds to water availability. Specifically, the land dominated by the annual life cycle of short-rooted herbaceous vegetation shows a relatively short CED compared to those covered by woody vegetation (Supplementary Fig. 3b). Our results show the same proportion of areas demonstrating increasing or decreasing trends over the past 3 decades. The increasing trends are mainly distributed in the wetter sub-humid areas where annual precipitation ranges from 800 to 1200 mm, where also the highest percentage of increasing sensitivity to water constraint was found (Supplementary Fig. 2a and Supplementary Fig. 3a). This could possibly be related to subtle changes in vegetation types in regards to their plant functioning or land management (Herrmann et al., 2020, Bennour et al., 2023).

### 4.2 Role of climate and climate variability in regulating vegetation-water response

By analysing the result of the RF model, our findings suggest that the temporal variability of CO_2_ concentration during the growing season can lead to varying effects on the trend of vegetation-water sensitivity. However, the CO_2_ data used here assumes a homogeneous CO_2_ concentration in the atmosphere (Lan, 2023). Hence, a possible mechanism behind the difference in CO_2_ variability could be attributed to the various start time and duration of the growing season. Besides that, another explanation could be the influence of elevated CO_2_. As concentrations of CO_2_ are rising during the same month each year, the essence of the increasing CO_2_ temporal variability is the increasing rate diversity in different months (Supplementary Fig. 4). Elevated atmospheric CO_2_ concentrations can influence vegetation by inhibiting the stomatal aperture (Ainsworth and Rogers, 2007, Pausata et al., 2020) thereby enhancing vegetation productivity (Wang et al., 2020, Zhang et al., 2022). This is referred to CO_2_ fertilization, which leads to the reduction of water loss and increased water use efficiency. As such, the elevated CO_2_ could indirectly decrease the vegetation-water sensitivity overall (Keenan et al., 2013). On the contrary, as vegetation productivity increases, it can result in an expansion of the area covered by leaves, subsequently causing an increase in water requirements (Tian and Zhang, 2020). In that situation, this demand may offset the advantage associated with CO_2_ fertilization, and thereby indirectly increase the water sensitivity (Zhang et al., 2022, Zhang et al., 2022, Zhang et al., 2022). Overall, the relationship between CO_2_ and vegetation-water sensitivity is complex and depends on many factors, including the specific plant species and environmental conditions. In the Sahel-Sudan region, an increased leaf area may have countered the expected decreasing trend in sensitivity associated with CO_2_ concentrations (Fig. 3b).

Temperature (mean and variability) are the second and third most important drivers of vegetation-water sensitivity. Previous research found that temperature strongly influences the vegetation's metabolism or photosynthesis (Akula and Ravishankar, 2011, Kläring and Krumbein, 2013, Hatfield and Prueger, 2015). The metabolism of vegetation would consume more water under increasing temperature, which may in turn intensify vegetation-water sensitivity. Hence, a warmer temperature or larger temperature variability stimulates increased vegetation-water sensitivity (Fig. 3c and d) by controlling the process of metabolism. Additionally, vegetation has a certain temperature optimum, which in cases where this threshold is exceeded might have adverse impacts on vegetation growth, thereby decreasing the vegetation-water sensitivity (Mathur et al., 2011).

Incoming solar radiation and temperature (mean and variability) are the top three drivers of CED based on the permutation importance result of the RF model. It is well-known that incoming radiation and temperature can significantly alter plant phenology by impacting the onset of the growing season and the length of the growing season (Cleland et al., 2007, Heumann et al., 2007). Furthermore, incoming solar radiation significantly impacts the Earth's temperature, and regions with higher incoming solar radiation generally exhibit higher temperatures compared to regions with lower solar radiation. Consequently, the influence of incoming solar radiation on vegetation growth can indirectly occur through its effect on temperature. Previous studies have indeed reported a pronounced increase in surface temperature in the Sahel-Sudan zone during recent decades (Engelbrecht et al., 2015, Iyakaremye et al., 2021). When the incoming radiation or temperature exceeds a certain threshold, as mentioned above, this results in damaged plant tissues (Mathur et al., 2011), which ultimately leads to a shorter water-related CED by exerting another control of the vegetation life cycle (Fig. 4b-d).

### 4.3 Limitations and implication for future work

Despite the reported progress in our understanding of the complex interplay between dryland vegetation growth and water availability, this work acknowledges the presence of certain limitations and uncertainties. First, since the CO_2_ data is not spatially explicit, there may be a bias between the CO_2_ ground truth and the CO_2_ data that impacts the accuracy of our attribution analysis. Second, the Mann-Kendall trend analysis only focuses on linear trends throughout the entire research period, which may neglect the potential effect of non-monotonic changes. Lastly, on the attribution analysis part, the influence from human management and other non-climatic effects (such as human density, irrigation, pollution, and land cover change) have not been considered, although other studies have shown that they may have a clear impact on the vegetation-water response (Wang et al., 2017, Horion et al., 2019).

## 5 Conclusion

This study provides insights into the mechanisms underlying vegetation response to water availability in dryland ecosystems, with implications for mitigating the potential impacts of future climate change. The research focused on observed changes in the Sahel-Sudan region, utilizing the Normalized Difference Vegetation Index (NDVI) and multi-timescale Standardized Precipitation Evapotranspiration Index (SPEI) as proxies for vegetation productivity and water constraint, respectively. We demonstrate notable spatio-temporal changes in the sensitivity of vegetation productivity to water constraints, along with variations in the cumulative effect duration (CED) of water constraints (the legacy effect of the impact of water constraints on vegetation) during the period 1982–2016. The trends in sensitivity among different types of land cover revealed a potential increasing threat of water constraints particularly in forest ecosystems of the region. We highlight the existence of diverse underlying mechanisms governing the complex interplay between vegetation productivity and water availability showing that changes in CO_2_, mean growing season temperature, and their temporal variability are primary drivers of changes in vegetation-water sensitivity. These results provides an improved understanding of the varying mechanisms of vegetation-water sensitivity observed across the Sahel-Sudan region being an important basis for safeguarding dryland ecosystems from the potential impacts of climate change and ensuring their long-term sustainability.

## Funding

This work was supported by the 10.13039/501100004543China Scholarship Council [grant number 20196400012]. R.F. Acknowledge support by the 10.13039/100008398Villum Foundation through the project ‘Deep Learning and Remote Sensing for Unlocking Global Ecosystem Resource Dynamics’ [DeReEco, grant number 34306] and by the 10.13039/501100004836Independent Research Fund Denmark [grant number 2032-00026B]. This work was also supported by the European Research Council (ERC) under the European Union’s Horizon 2020 research and innovation programme [grant number 947757 TOFDRY] and a DFF Sapere Aude [grant number 9064-00049B].

## CRediT authorship contribution statement

**Zhang Wenmin:** Methodology, Writing – review & editing. **Abel Christin:** Writing – review & editing. **Horion Stéphanie:** Formal analysis, Writing – review & editing. **Lu Tingting:** Conceptualization, Methodology, Writing – original draft. **Brandt Martin:** Writing – review & editing. **Huang Ke:** Conceptualization. **Fensholt Rasmus:** Supervision, Writing – review & editing.

## Declaration of Competing Interest

The authors declare that they have no known competing financial interests or personal relationships that could have appeared to influence the work reported in this paper.
