# Supplementary material for: Changes in vegetation-water response in the Sahel-Sudan during recent decades
Source: J Hydrol Reg Stud. 2024 Apr;52:101672. doi: 10.1016/j.ejrh.2024.101672 (PMC10993624; doi:10.1016/j.ejrh.2024.101672)
Supplement: Supplementary file 1 — Supplementary material. [file mmc1.docx]

Supplementary figures:


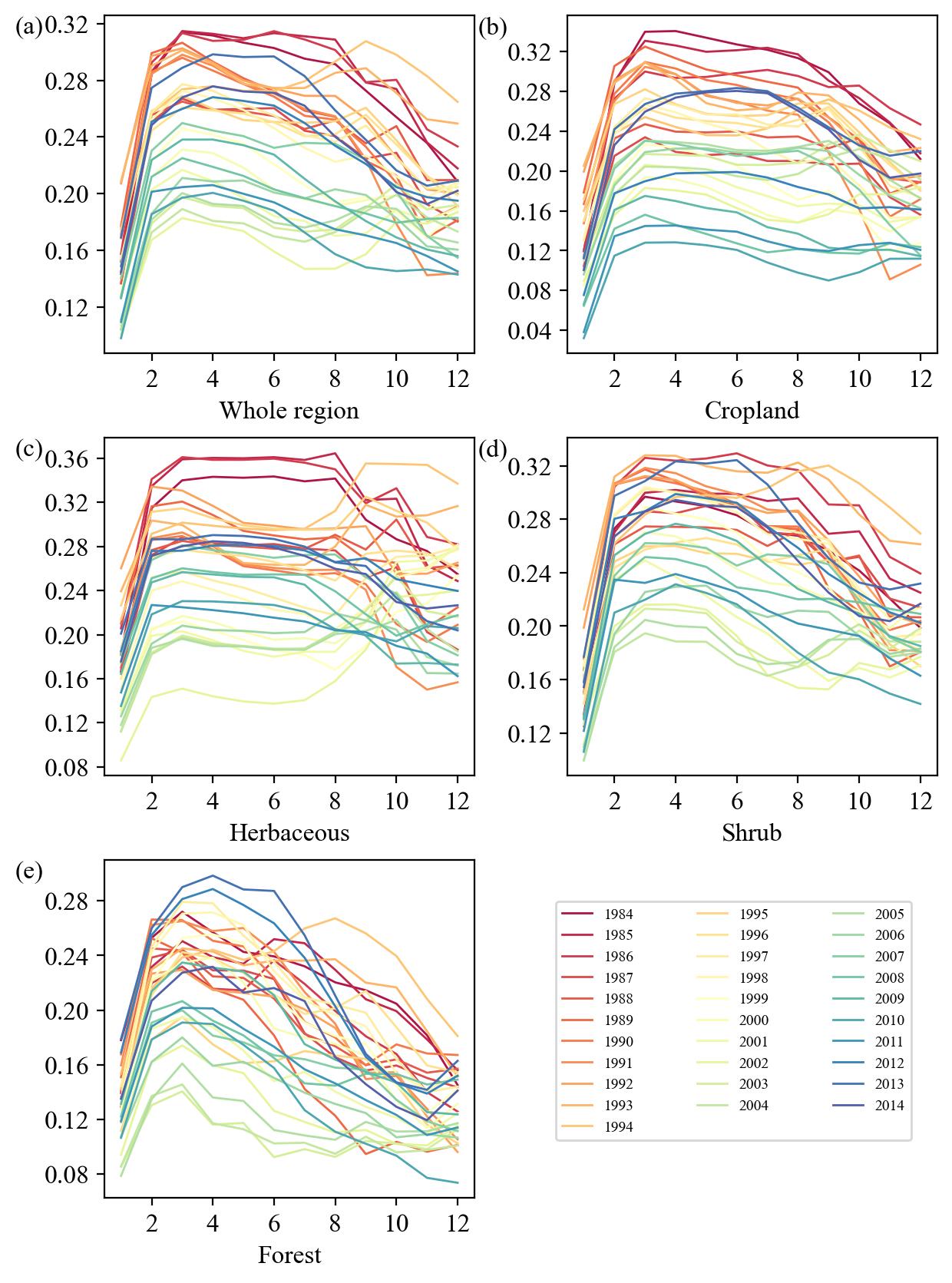


Fig. 1. (a) The average r-value between NDVI anomalies and SPEI at time scale from 1 to 12 months for different years in the Sahel-Sudan region. (b)-(e) same as (a) but for different land cover types: cropland, herbaceous, shrubs, and forest.


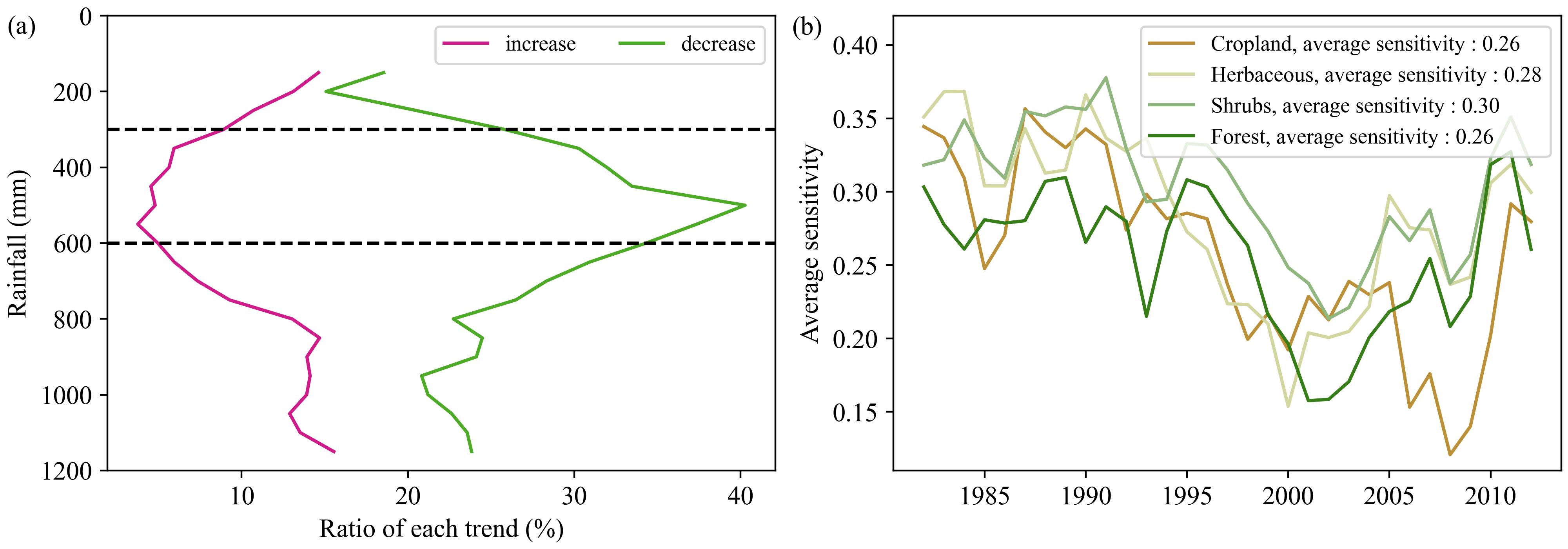


Fig. 2. (a) The ratio of each sensitivity trend group along the precipitation gradient (50 mm steps). The horizontal dashed lines represent 300 mm and 600 mm annual rainfall, which indicate divisions between arid, semi-arid, and sub-humid zones. (b) The temporal dynamics of average sensitivity for different land cover types in water constrained region.


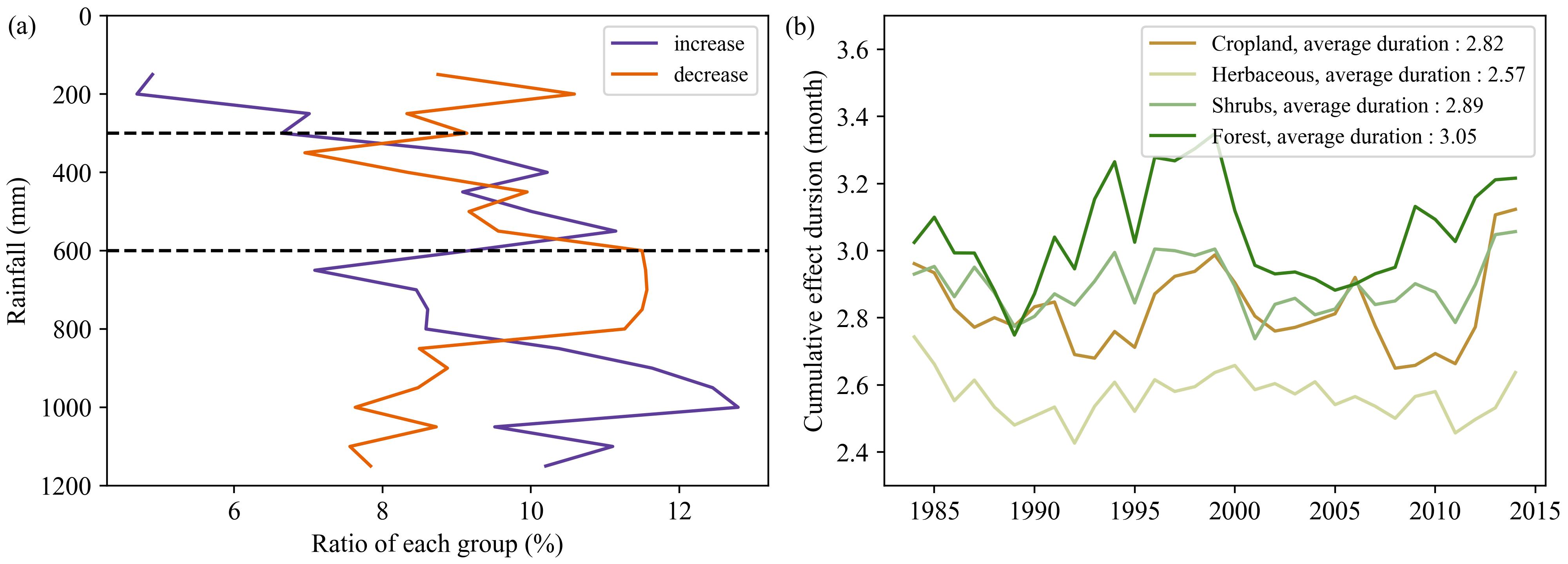


Fig. 3. (a) The ratio of increased/decreased trend of CED along the precipitation gradient (50 mm steps). The horizontal dashed lines represent 300 mm and 600 mm annual rainfall, which indicate divisions between arid, semi-arid, and sub-humid zones. (b) The temporal dynamics of CED for different land cover types in water constrained regions.


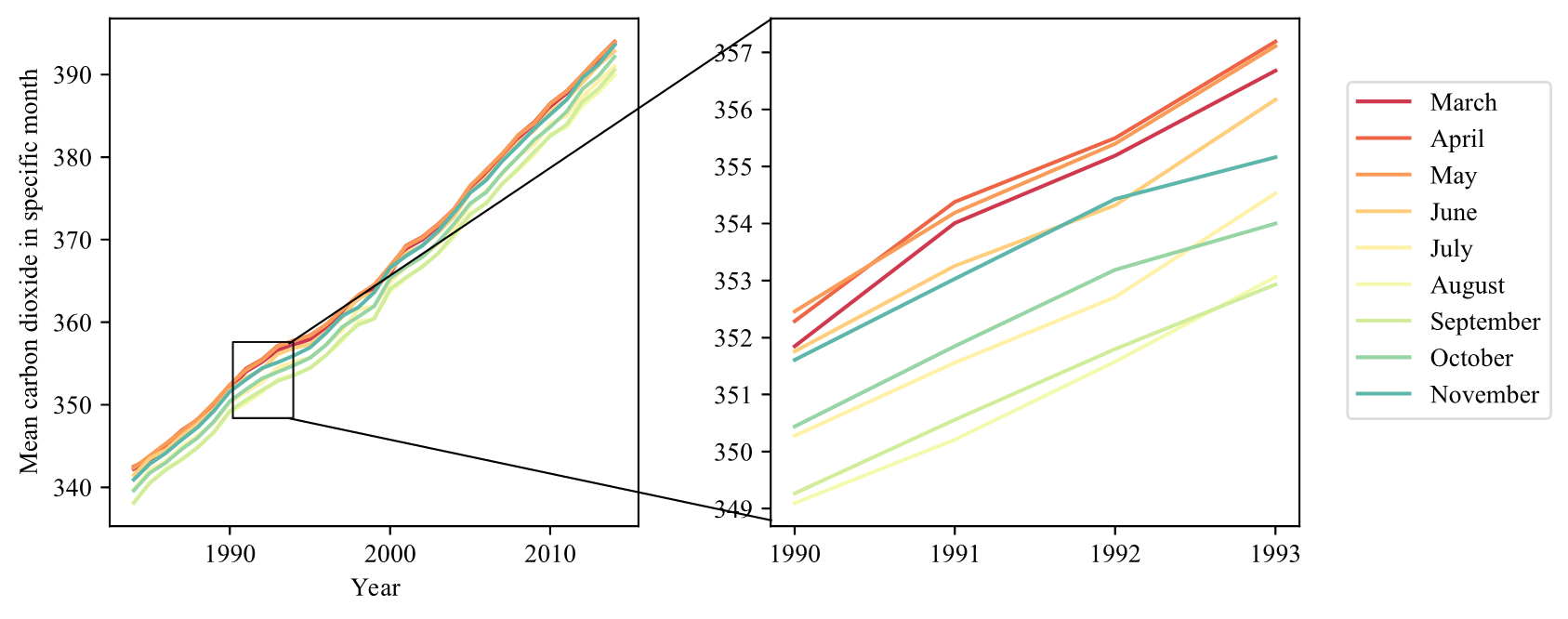


Fig. 4. The average CO_2_ concentration in the different months encompassing the growing season in the Sahel-Sudan region over the period of analysis. The right-hand side zoomed-in figure is limited to the year 1990 to 1993.
